# Wear Performance of Circular Shim against Cam in Engine Bench Test

**DOI:** 10.3390/ma15186293

**Published:** 2022-09-10

**Authors:** Cheng-Di Li, Jing-Si Wang, Xu Han, Feng-Ming Du, Geng-Shuo Liu, Ren-Jin Lin

**Affiliations:** 1 National Center for International Research of Subsea Engineering Technology and Equipment, Dalian Maritime University, Dalian 116026, China; 2 Xinyu Key Laboratory of Materials Technology and Application for Intelligent Manufacturing, Xinyu University, Xinyu 338004, China

**Keywords:** shim, cam, wear performance, engine bench test

## Abstract

Unlike the conventional engine, the valve train of a certain type of engine uses a circular shim instead of a tappet to wear against the cam. To verify the reliability of the shim, an engine bench test was used to test its wear performance. The total duration of the bench test was 1000 h, which was divided into three stages. In each stage, the test equipment was stopped, and the shims were disassembled to observe the surface morphology during the worn process. Precious long-term data were obtained. With the extension of the bench test time, weight loss increased. The maximum weight loss occurs 1000 h after worn, which is about twice that of 350 h. During the wear process, a plastic flow of material was found on the subsurface, and fatigue wear marks occurred on the surface. With an increase in test time, the wear marks increased, leading to material spalling and the formation of pits. The wear mechanism was the mixed wear of fatigue wear and adhesive wear.

## 1. Introduction

The valve train is an important part of the engine. It regularly opens and closes the intake and exhaust valves of each cylinder so that air can enter the cylinder in time and exhaust gas can be discharged smoothly. In the valve train, the friction pair where the cam is located is easy to wear. Even a slight change in geometry will lead to the deterioration of stress distribution and affect the service life of the cam friction pair. It seriously affects the transmission and geometric accuracy of the valve train, thus causing compression loss and energy loss of the engine, and seriously reducing the output power of the engine. Therefore, it is of great significance to research the wear performance of the valve train to prolong the service life of the engine, save energy, and reduce emissions.

**Figure 1:**
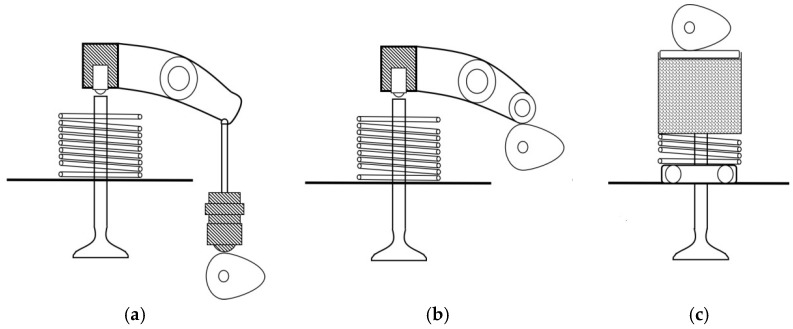
Types of Camshaft Arrangements: (**a**) down-mounted camshaft, (**b**) middle-mounted camshaft; (**c**) up-mounted camshaft.

As an important friction pair of the valve train, the cam is located on the camshaft. There are great differences in the camshaft positions of the valve train in the engine, which can be divided into three types according to the different camshaft arrangements [1]. The three types are down-mounted camshaft, middle-mounted camshaft, and up-mounted camshaft (Figure 1). The down-mounted camshaft is located inside the crankcase and is directly driven by the crankshaft. Its advantage is that it is close to the crankshaft, but its disadvantage is that it has a long transmission distance, requires many parts, and the rigidity of the whole valve train is poor. When the engine is running at high speed, it may destroy the motion law of the inlet valve and exhaust valve, and then affect the normal opening and closing of the valve. Therefore, a down-mounted camshaft is usually used in low-speed engines. Compared to the down-mounted camshaft, the length of the push rod of the middle-mounted camshaft is reduced. Tappet can be used to directly replace the push rod, which reduces the reciprocating mass of the valve train and increases the stiffness of the valve train. The middle-mounted camshaft is suitable for higher-speed engines. However, for high-speed reinforced engines, an up-mounted camshaft is more suitable [2]. The camshaft is located above the cylinder and directly participates in driving the valve’s opening and closing. Its advantage is that the push rod, rocker arm, and other parts are eliminated. In this way, the stiffness of the valve train is increased, and the vibration is reduced at a high speed. In most engines with an up-mounted camshaft, the cam contacts the tappet when it rotates to control the opening and closing of the valve. Therefore, the cam and tappet as friction pairs have been studied by some researchers [3,4,5].

Wang G.H. analyzed the failure mechanism of tappets in the valve train of a high-speed and heavy-load diesel engine [6]. To reduce the failure rate of cam/tappet, adding coating on the surface is a way to enhance the wear resistance. B.G. Lyu presented wear resistance by surface coating on cam/tappet pairs under different conditions [7]. M. Marian studied friction reduction of cam/tappet by surface modifications [8]. D. Ozkan reported the wear behavior of various coatings on cams and tappets, such as Chromium nitride-coated copper beryllium, TiB thin film-coated, TiAlN, AlTiN, and AlCrN Coatings [9,10,11]. Y. Al-Jeboori studied the rotation of coated tappets at different temperatures [12]. Coating enhances the strength of the material’s surface. In addition to the influence factor of surface strength on wear behavior, lubrication is also an important influence factor. Several researchers have committed to the research of lubricating oil between cam and tappet. R. Zahid presented the effect of lubricant additives on the wear performance of cam and tappet [13,14]. H.W. Tang, P. Singh, and S. Poonia analyzed the oil film in the wear process in detail [15,16,17]. Due to the complexity of lubrication, some calculation models can better explain friction mechanisms. X.H. Meng established an EHL model [18]. M. Marian et al. studied friction reduction in EHL contacts by surface microtexturing—tribological performance [19,20]. Most of the above wear tests are carried out through component tests or material tests rather than engine bench tests. The contact stress between the cam and tappet is affected by cylinder pressure, which has the characteristics of alternating and large peak values. If only external pressure is applied, the actual peak value is often not reached. Therefore, a bench test is needed to test the reliability of the cam and tappet, but the bench test cost is high. Many scholars use the method of establishing dynamic models to simulate the wear process, such as K. Siczek, C. Orgeldinger, and B. Hu [21,22,23,24].

**Figure 2:**
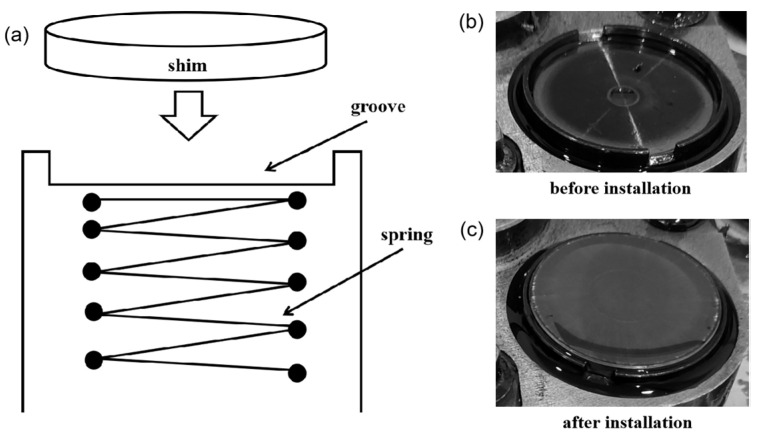
The circular shim installation: (**a**) installation diagram, (**b**) before installation; (**c**) after installation.

In the cam–tappet friction pair, the tappet is usually worn more than the cam, and the cam is slightly worn. In the case of serious wear, the tappet needs to be replaced in time. Otherwise, serious wear of the tappet will affect the normal operation of the valve train and cause engine failure [25]. However, the removal and replacement of the tappet is cumbersome. A circular shim is designed for a certain engine to replace the tappet, which makes disassembly easier. As shown in Figure 2, the shim is installed in the groove, and the disassembly is simple. The size of the circular shim is smaller than that of the tappet, with a lower cost and more convenient processing. The contact form between shim and cam is line contact, and the motion law is pulse type. When the base circle of the cam faces the shim, the shim is not under force. As the cam rotates, the cam tip presses against the shim, which transmits the pressure to the valve for opening. During the friction process, the shim is also subject to the side thrust brought about by the rotation of the cam, making it rotate slightly in the circumferential direction. It is theoretically feasible to apply this kind of shim to the valve train of the engine, but whether the application effect is excellent or not must be verified by the engine bench test. The bench test is an important method for testing the reliability of engine parts, which simulates the real working conditions and can obtain the wear conditions close to the actual engine. Most researchers often use computational simulation for cam friction pairs, and bench tests are rarely reported. This is due to the higher cost and longer time spent during bench tests. At present, the wear mechanism of shim is unclear. It is necessary to evaluate the wear performance of shim through a bench test. In this paper, the shim was worn against the cam during the bench test, and the total test time was about 1000 h. It is precious to obtain wear test data for such a long test time. The worn surface morphology of shim was obtained, and the wear mechanism was analyzed.

## 2. Experimental

**Figure 3:**
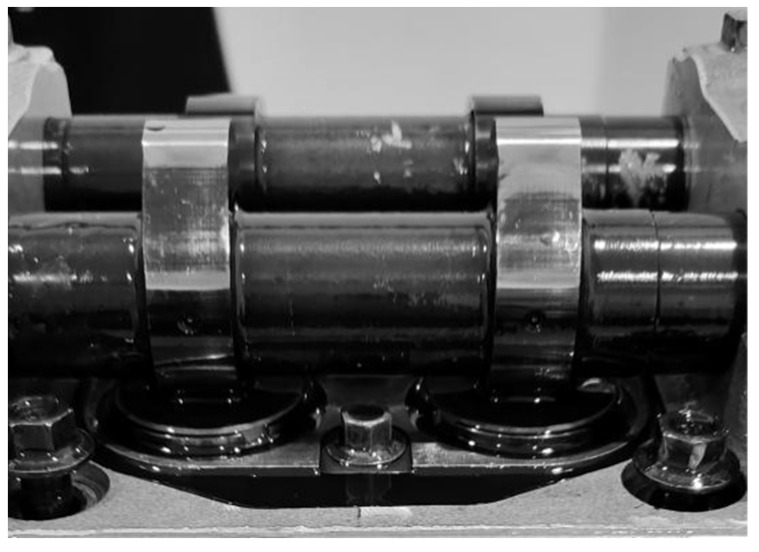
Contact state of the cam and shim.

The thickness of the circular shim is 4 mm, and its diameter is 41 mm. The material of shim is GCr15 steel, and the chemical element content is presented in Table 1. The material of the cam is 45 steel, with a height of 52.64 mm. The hardness of the cam is 60 HRC. The camshaft, spring, valve, cylinder head, and other parts in the bench test are consistent with those used in the real engine. The engine in the bench test has 12 cylinders; each cylinder has 4 cam-shim friction pairs, a total of 48 friction pairs. During the engine bench test, the speed is 1900 r/min, and the torque is 5000 N·m. The operating condition for testing is that the maximum torque load spectrum of this type of engine is loaded with constant power. The load and torque are larger values in actual vehicle use. The tests were performed lubricated with 15 W-40 oil (CF-4). The contact state of the cam and shim is shown in Figure 3. When the base circle of the cam faces the shim, they do not contact them, and there is a gap between them. The length of the gap is 0.68 ± 0.03 mm. At this time, the valve is closed. When the cam rotates, the tip of the cam squeezes the shim, and the valve opens.

The total duration of the engine bench test is 1000 h, which is divided into three stages (350 h, 350 h, 300 h). Therefore, the wear time to each stage is 350 h, 700 h, and 1000 h. The worn surface morphologies were analyzed using optical microscopy (OM) and scanning electron microscopy (SEM). Several wear loss measurement methods are available. The amount of wear can be characterized by wear depth, wear area, wear volume, or weight loss. Among them, the method of weight difference is more convenient to measure. Other methods are not applicable to the shim because the boundary between worn and unworn areas is not very clear, and the measurement is easily inaccurate. Therefore, the weight loss of shim is measured after shutdown in each stage. The weight difference before and after wear in each test stage represents weight loss. In addition, some scholars have proposed more insight-based measurement methods. The deformation evolution during the nanoindentation process is evaluated using the quasi-static method [26]. A parametric study using an analysis of variance technique was used to efficiently determine the influence of the deformation path and size scale with respect to yield and plastic energy [27]. Although advanced measurement methods are not used in this paper and more convenient methods are selected, a better characterization method will be employed in future studies.

## 3. Results and Discussion

### 3.1. Worn Surface

**Figure 4:**
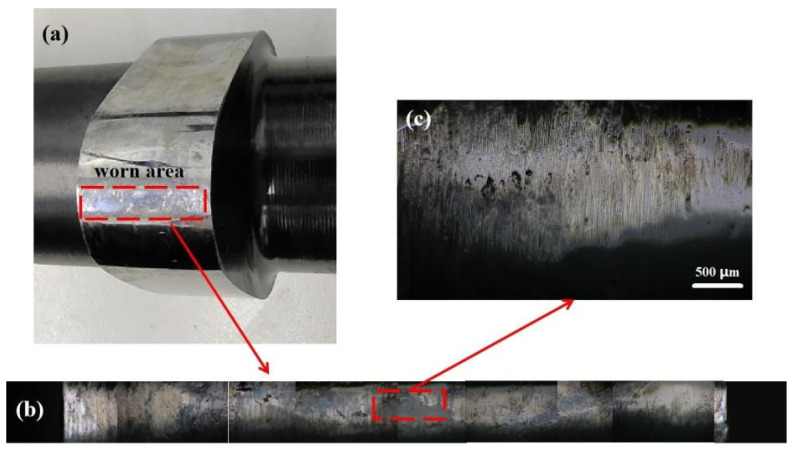
The worn area of the cam: (**a**) macro photo, (**b**) surface morphology of stitching after continuous shooting with optical microscope, (**c**) amplification of the middle position of the wear area.

The worn area of the cam is shown in Figure 4. The wear behavior mainly occurs at the tip of the cam (Figure 4a). For convenience of observation, the wear area is continuously photographed with an optical microscope (Figure 4b). The most severely worn place is the middle position. There are many longitudinal scratches in this position (Figure 4c). This is because when the cam rotates, the tip first contacts the shim, and then continuously pressurizes until the tip leaves the shim. The cam always rotates in one direction, so the wear marks show regular longitudinal scratches.

**Figure 5:**
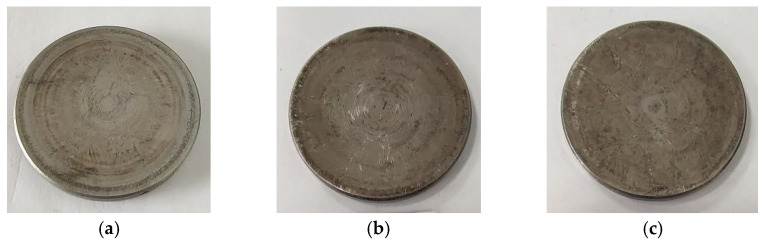
The macro photos of shims after the engine bench test: (**a**) 350 h, (**b**) 700 h, (**c**) 1000 h.

**Figure 6:**
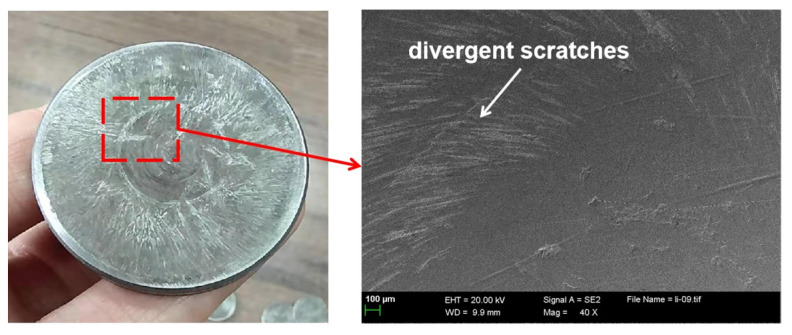
The worn morphology is near the center of the shim.

Figure 5 shows macro photos of shims after the engine bench test. Divergent scratches can be clearly seen on the shim surface worn for 350 h in the circumferential direction (Figure 5a). The wear marks of shim worn for 700 h were deepened (Figure 5b). The surface of the shim worn for 1000 h is rough, and the surface is covered with similar wrinkled wear marks (Figure 5c). With the extension of wear time, the wear of shim becomes more serious. Figure 6 presents the worn morphology near the center of the shim. The wear trace at the center of the shim is slight. Divergent scratches are found at a distance from the center of the circle, extending to the edge.

**Figure 7:**
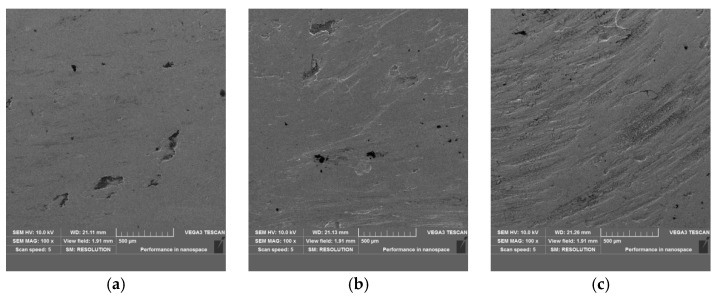
The worn surface morphology of shims after engine bench test (SEM): (**a**) 350 h, (**b**) 700 h, (**c**) 1000 h.

Figure 7 shows the surface morphology of the worn area of shims. The surface worn for 350 h was smoother with fewer scratches than the surface worn for 700 h (Figure 7a). The worn marks increased after being worn for 700 h, and the potholes left by material falling off increased (Figure 7b). When worn for 1000 h, the surface plastic deformation was obvious, with deeper and more worn scratches (Figure 7c).

**Figure 8:**
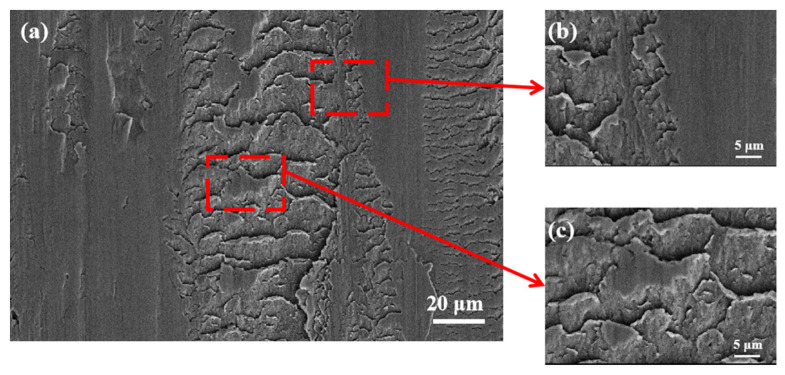
The worn marks on the surface of shim: (**a**) wrinkled worn marks, (**b**) amplification of the edge of the worn marks, (**c**) amplification of the worn marks.

It can be seen from Figure 8 that the worn marks caused by the cam rolling on the shim surface produced serious plastic deformation. Obvious material cracking due to fatigue wear can be observed at the edge of the worn marks (Figure 8b). The plastic flow of surface materials under the action of rolling pressure causes local accumulation (Figure 8c), and the surface damage is very serious at this time.

**Figure 9:**
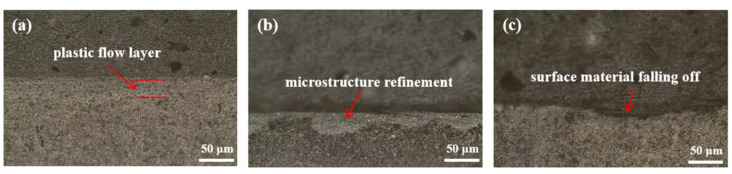
The cross-section of the worn morphology of shims after engine bench test (OM): (**a**) 350 h, (**b**) 700 h, (**c**) 1000 h.

Figure 9 shows a cross-section of the worn morphology of shims after the engine bench test. From the worn section for 350 h (Figure 9a), it can be seen that there are signs of plastic flow on the subsurface, forming a deformation layer with a thickness of 20 μm. When the worn time was 700 h, microstructure refinement appears on the subsurface with more fine white carbides (Figure 9b). This is the phenomenon of machining refinement caused by the long-term rolling of the cam. However, with the worn time extending to 1000 h, the subsurface plastic deformation was serious, resulting in the surface material falling off and leaving pits (Figure 9c). This was due to the shear force destroying the subsurface of the shim, and metal adhesion occurred on the contact surface. Subsequently, during the rolling of the cam, the adhesion position was damaged, and the surface was scratched and even peeled off. Therefore, there was not only fatigue but also adhesive wear during the wear process.

### 3.2. Weight Loss

**Figure 10:**
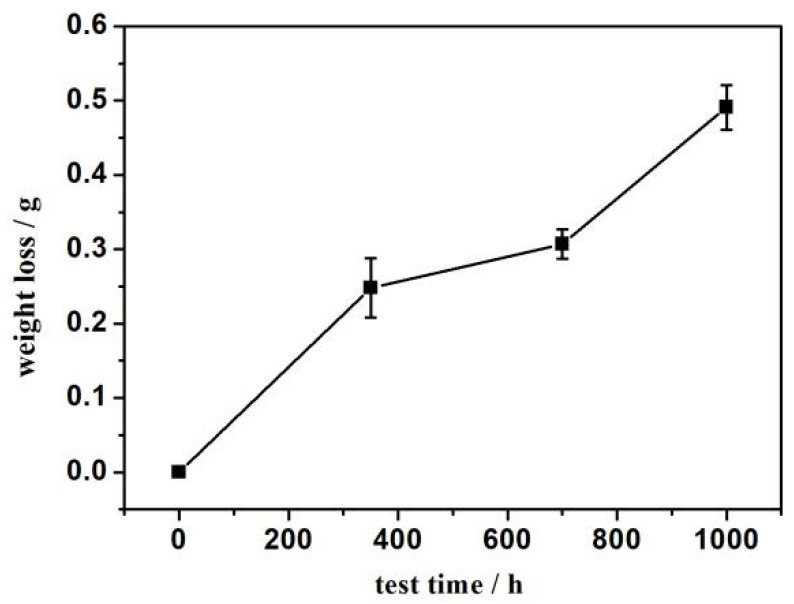
Weight loss of shim.

The weight loss of shims is presented in Figure 10. The weight loss of shim after worn for 350 h is 0.248 g, that after worn for 700 h is 0.307 g, and that after worn for 1000 h is 0.491 g. With the extension of the bench test time, weight loss increased. The maximum weight loss occurs 1000 h after worn, which is about twice that of 350 h. The running-in wear mode occurred before 350 h. When the worn time was 350~700 h, the wear entered a steady-state wear stage, and the weight loss increased slowly at this time. When the worn time was 700~1000 h, the wear tate increased greatly, and at this time, it was in accelerated wear.

### 3.3. Wear Mechanism

**Figure 11:**
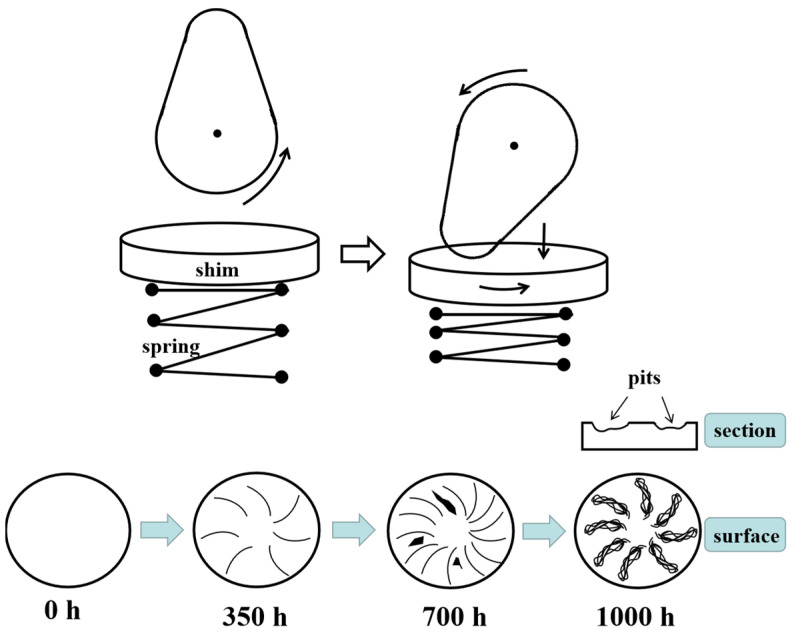
Schematic illustration of the wear process of shim against cam.

The wear process of shim against cam is shown in Figure 11. When the cam base circle faced the shim, the two did not contact, and the shim was not stressed. When the cam rotated to the tip to contact the shim, the cam rolled the shim and compressed the spring due to pressure. Under the action of the side thrust of the cam rotation, the shim rotates slightly in the circumferential direction. Therefore, the wear marks on the shim surface appeared to diverge from the center to the edge of the shim. When the worn time was extended to 700 h, the wear marks increased. Under the continuous rolling of the cam, the fatigue wear of the shim surface was aggravated, and the plastic deformation of the material surface was serious, even peeling off. After being worn for 1000 h, the plastic deformation of the shim intensified, and the wear marks of material accumulation appeared on the worn surface. The worn marks proved the existence of fatigue. At this time, there were pits in the worn position of the shim. The contact between the cam and the shim is subject to alternating loads, and the contact stress is large. Fatigue wear marks will occur during wear. With an increase in test time, the wear marks will further develop, leading to material spalling and the formation of pits. The weight loss of shim increased due to material spalling. In addition, when the cam wore against the shim, the shear force destroyed the subsurface of the shim, and metal adhesion occurred on the contact surface. Subsequently, during the rolling of the cam, the adhesion position was damaged, and the surface was scratched and even peeled off. Therefore, fatigue wear and adhesion wear occur during the wear process.

The wear-resistant coating on the shim may reduce wear in the tests. For example, DLC coatings are used on tappets to enhance wear resistance. This requires researchers to conduct research in the future.

## 4. Conclusions

The wear performance of circular shim against the cam in the engine bench test has been discussed in this paper. The total duration of the bench test is 1000 h, which is divided into three stages. In each stage, the test equipment was stopped, and the shims were disassembled to observe the surface morphology during the worn process. The important conclusions that emerge from this paper are as follows:

(1) With the extension of the bench test time, weight loss increased. The maximum weight loss occurs 1000 h after worn, which is about twice that of 350 h.
(2) When the cam base circle faced the shim, the two did not contact, and the shim was not stressed. When the cam rotated to the tip to contact the shim, the cam rolled the shim and compressed the spring due to pressure. The shim rotates slightly in the circumferential direction. Therefore, the wear marks on the shim surface appeared to diverge from the center to the edge of the shim.
(3) During the wear process, a plastic flow of material was found on the subsurface, and fatigue wear marks occurred on the surface. With an increase in test time, the wear marks increased, leading to material spalling and the formation of pits. The wear mechanism was the mixed wear of fatigue wear and adhesive wear.

## Figures and Tables

**Table 1:** Chemical element content of GCr15 steel.

Element	C	Si	Mn	Cr	Mo	P	S	Ni	Cu
Content/%	0.95	0.15	0.25	1.40	≤0.10	≤0.025	≤0.025	≤0.30	≤0.25

## Data Availability

Not applicable.
